# Impacts of consumption tracking and tailored feedback on meeting nutritional recommendations: a longitudinal regression discontinuity study

**DOI:** 10.1186/s12937-025-01149-x

**Published:** 2025-05-23

**Authors:** Nathaniel Jensen, Watson Lepariyo, Vincent Alulu, Simbarashe Sibanda

**Affiliations:** 1 University of Edinburgh, Edinburgh, UK; 2 International Livestock Research Institute (ILRI), Nairobi, Kenya; 3 University of California-San Diego, San Diego, CA USA; 4 Food, Agriculture and Natural Resources Policy Analysis Network (FANRPAN), Pretoria, South Africa

**Keywords:** Consumption behavior, Nutrition information, Regression discontinuity

## Abstract

**Background:**

Malnutrition continues to have large and negative impacts on millions of people. Lack of nutrition education and access to accurate information can be large barriers to healthy eating.

**Methods:**

In this paper, we causally tested if providing participants with consumption tracking information accompanied by tailored messaging that referenced internationally recognized dietary guidelines improved their consumption patterns. To do so, we developed a smartphone application that participants used to record their consumption and that of their children. Those self-recorded data were then used to provide the participants with tailored feedback by comparing their recorded consumption against recommended consumption patterns. The causal impacts of the tailored feedback were estimated using a regression discontinuity estimation strategy and validated using alternative empirical strategies and a parallel dataset collected from the same participants by Community Health Volunteers.

**Results:**

We found that the informational and feedback treatments improved consumption patterns of the caregivers and their children. Specifically, once caregivers began receiving tracking information and tailored feedback on their children’s diet, their children’s likelihood of meeting the minimum dietary threshold increased by at least 23 percentage points. An analogous, although smaller and less precisely estimated, effect on the caregivers’ consumption was caused by providing them with tracking and feedback information on their own consumption. To verify these findings, we tested for the same effects using a parallel dataset collected by Community Health Volunteers from the same participants at the same period. The results of these analysis remained consistent with those estimated from self-recorded data but showed smaller effect sizes. Tests for persistence of the effects found no loss in impacts over the remaining months of the project.

**Conclusions:**

These findings show that improving access to information on recommended consumption and providing easy methods for tracking own performance against those recommendations can improve consumption patterns while also demonstrating that low-cost, light-touch approaches can be effective for collecting related data and delivering such services.

**Trial registration:**

Pan African Clinical Trial Registry ACTR202407500217236. Retrospectively registered on July 15 2024.

**Supplementary Information:**

The online version contains supplementary material available at 10.1186/s12937-025-01149-x.

## Background

Malnutrition and poor diet continue to be one of the main challenges that society faces and no region of the world meets the recommendations for healthy diets [1, 2]. Undernutrition increases the likelihood of mortality and disease [3]. For example, it is estimated that poor diets led to over 12 million deaths in 2018 alone [2]. Africa has the world’s highest average rate of undernourishment [4] and the dryland regions of Africa often face the highest rates of child malnutrition in the continent [5–7].

A lack of nutritional knowledge and information can be a constraint to healthy eating. For example, studies across a variety of contexts have found that providing education on recommended consumption patterns can improve consumption behavior (e.g., in Malawi [8], in the US [9], in Australia [10]). Information gaps can also exist in one’s perceptions of their own behavior. For example, several studies find that exercise tracking applications can increase exercise [11, 12], can improve the efficacy of weight loss programs [13] and can improve health consciousness and perceived physical health [14]. While there are many candidate mechanisms by which such tracking could impact behavior, there are two that are especially relevant for this research. The first is that the tracking processes employed (e.g., pedometers, smart phone applications) ease the participants’ burden of accurately tracking their own behavior, thereby improving the likelihood that participants have accurate information on their own behavior. For example, Gustafson and Zeballos found that providing a simple automated summation of calories while ordering online food can reduce the number of calories ordered and increase the accuracy of participants’ accuracy in estimating the number of calories that they ordered [15]. The second mechanism is that the presentation of tracked behavior makes it easier for participants to observe their progress against recommendations, which then motivates greater effort to meet those recommendations, while also making them more salient. Evidence confirms that adding performance feedback to tracking significantly boosts effectiveness [16], and the combination of tracking and feedback have been shown to be effective in changing consumption behavior in several settings [17, 18]. In a study similar to that performed by Gustafson and Zeballos, VanEpps et al., found that providing traffic light labeling (i.e. reg, green, yellow) that reflected dietary guidelines along-side aggregated calorie counts further reduced calories ordered [19]. While many informational and feedback interventions have been successful in changing behavior in some settings, there are also many studies that find that they have no impacts. Systematic reviews studying these types of interventions highlight that context, user experience, and stakeholder engagement are likely to affect their impact. (e.g., [20, 21]).

This research examined whether providing caregivers with improved access to performance tracking and feedback about their own and their child’s nutrition, would improve adherence to recommended dietary behaviors. We developed *Mbiotisho,* a smartphone application that enabled caregivers to record health and nutrition data on both themselves and their children.^1^ The application processed the caregivers’ submissions in real-time and provided them with simple up-to-date tracking metrics (diet surveillance) and tailored recommendations (diet education) by comparing their behavior against internationally recognized standards for consumption. We used the caregivers’ self-recorded consumption data and data collected in parallel by a 3rd party, to test for the causal impacts of these features on participants’ consumption. Causal identification of the feature was established by taking advantage of a delayed rollout of the tracking and feedback features. We found that the tracking and feedback features improved consumption by children but that the evidence was mixed for their caretakers, especially among food groups that were already consumed at relatively high frequency.

While prior research has established the potential behavioral effects of tracking and feedback on consumption in some settings, this study provides the first evidence of their efficacy in the rural development settings using a low-cost scalable technology. Specifically, our study takes place in the remote pastoral regions of Samburu County in northern Kenya, where malnutrition rates are high, literacy and cellular connectivity rates are extremely low, and where there is little nutritional knowledge In such settings, nutritional education, monitoring, and recommendations currently require in-person visits, which, while extremely important, are also often cost prohibitive; meaning that they rarely take place. This research shows that participants do change behavior when provided with the feedback features provided by this project and, therefore, that the application was effective in filling some of the information gaps that these households face.

## Methods

### Study design and implementation

This research used data from the study titled “Improving Dietary and Health Data for Decision-Making in Agriculture and Nutrition Actions in Africa”, which developed a smart phone application that was used by individuals to record information related to several indicators, including consumption and morbidity of themselves and their children. In October of 2019, the study recruited caregivers from four community health units in Samburu County, Kenya. The participants were randomly selected from a stratified roster of eligible caregivers that were being served by Community Health Volunteers (CHV) in those four community health units.

Inclusion criteria for participation were as follows.

1. The individual was currently a primary caregiver for a child between 6 and 48 months.
2. The individual was already working with one of the 18 CHVs that the local Community Health Extension Worker designated to work on the project.
3. The CHV did not have information that the caregiver was about to move outside of the county.

The selected caregivers and their spouses were invited to an informational session in their region and then invited to participate in the project. Agreeing to participate meant completing a consent form that included separate sections for themselves and for their youngest child over six months old, and for receiving project property—the smartphone and solar chargers—for their use during the project.

The study recruited 189 caregivers. The participants were trained on how to use smartphones, nutritional concepts, including food groups and suggested consumption for them and their children, and the application, which did not require literacy or numeracy. The trainings were done in series in the four different locations by the same trainers. Each training took 9 days, which included 3 days of home practice by the caregivers. Caregivers could begin recording measurements after their final day of training. For the remainder of the project period, participants were incentivized to use the application to record a variety of behaviors and conditions.^2^ In this study we focused on self-recorded consumption of the 16 food groups described in FAO guidelines [23] using 24-h recall. The caregivers recorded separately for themselves and the child participant. For more details on the smart phone application, sampling, and the project implementation, please see Jensen et al. (2023).

Because the trainings were done in series, the first day of data collection was staggered by training location, ranging from October 30, 2019 to November 30, 2019. The implementation lasted 13 months. During that period, the participants recorded and submitted over 60,000 reports on their consumption and that of their participating child. During this 13-month period, CHVs also collected data each month from the participants and their children on the same indicators, including 24-h recall consumption by food group, that the participants were recording and submitting. The data were collected by the CHVs while they performed their monthly visits to the caregivers. The 24-h consumption data used in this study were collected by asking the caregiver about her consumption in the last 24 h and then asking the same set of questions to the caregiver about the participating child.

These parallel data collected by the CHVs were used to verify the findings of analysis generated from the data collected by caregivers.

**Fig. 1:**
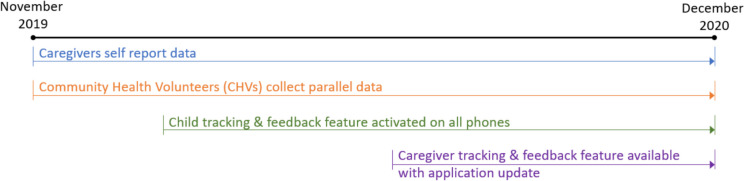
Timeline of project events

Figure 1 illustrates the project timeline, including the two data collection activities—recording by caregivers and parallel data collected by CHVs—and the intervention timing, which we describe in the next section.

#### Intervention

**Fig. 2:**
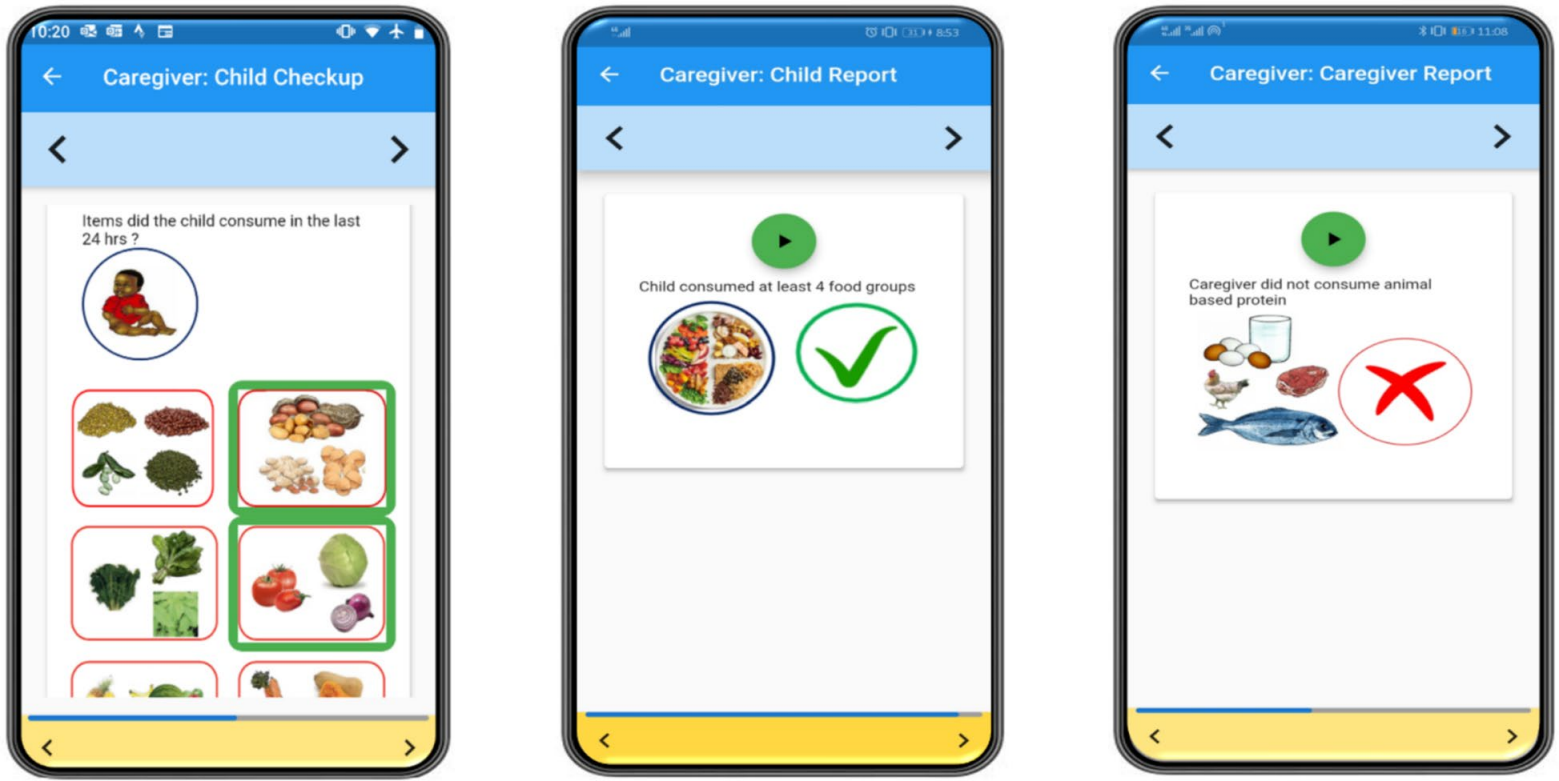
Example of the process for recording child consumption (left panel), feedback on child consumption (middle panel), and feedback on caregiver consumption (right panel)

To test for impacts of improved consumption information, we developed a monitoring and feedback feature in the smartphone application that participants were using as part of the project. This feature processed recent data recorded by the participant, and then provided them with customized messaging on their progress against benchmarks. The messages were communicated through a series of screens, each with images and audio in the local language. Each message described the recommended benchmarks and a simple assessment of the records submitted by the caregiver. The first feedback process focused on the participating child and provided the age-appropriate recommendations by the WHO [24] on daily breastfeeding frequency and dietary diversity (example provided in Fig. 2, middle panel^3^). The second feedback process on the caregiver was later launched. This process recommended that the caregiver consume *animal source foods*, *dark green leafy vegetables*, and Vitamin A rich fruits and vegetables (*orange fleshed fruits and vegetables*) each day and reported the caregivers’ own records on these three food groups (example provided in Fig. 2, right panel).

To test the impact of the monitoring and customized feedback feature, we delayed the release of the child feedback feature until December 15 th, 2019, six weeks after data collection had started (Fig. 1). As stated above, this original feature only provided feedback on information submitted about children. The project participants responded positively to that original feature and requested that feedback on the caregivers’ reports be added as well. In response, the application was updated to include information on the caregivers’ consumption. The updated application was launched on June 1, 2020 (Fig. 1). The field staff sent messages to all caregivers with instructions to update their application and CHVs were asked to reach out to their clients and assist those that reported challenges with the update process. The database tracked if and when individuals updated their software.

There are three fundamental differences in the launch of this second caregiver-focused feature from the launch of the first feature. First, the launch was done much later in the project so that there was a longer period and more observations before it launched. Second, the launch of the second set of features required that the participants manually update the software, a process that is observable in the data collected by the application. The result was considerable variation in the date that caregivers updated their application. In some cases, the update did not happen until many weeks after the new feature was launched and, in a few cases, it never happened at all. Third, the updated app also logged when the feedback features were opened, so that we could observe when the feedback was accessed.

These three changes are important because they created observable variation in treatment status across the sample — starting on June 1, 2020. On any particular day, there were participants that had updated their software and accessed the caregiver-tracking feature and those that had not. We return to this detail in the section on empirical strategies.

#### Outcomes of interest

We tested the impact of the initial feedback feature on the likelihood that the child met the Minimum Dietary Diversity Score for Children (MDD-C), which requires eating at least four food groups from a seven- food group classification developed by WHO [24], and the likelihood of women eating from three food groups— *animal source foods*, *dark green leafy vegetables*, *orange fleshed fruits and vegetables*—selected for their importance for women of reproductive age [25]. Each indicator was calculated as a binary variable for each 24-h recall observation, where the value of one indicates that the consumption target was met, and zero indicates that it was not met.

#### Ethical clearance

This study received ethical clearance from the Institutional Research Ethics Committee at the International Livestock Research Institute (Ref no: ILRI-IREC2019-15) and the National Commission for Science, Technology and Innovation of Kenya (Ref: No. NACOSTI/P/19/72940/31932).

### Empirical strategies

We employed two empirical strategies to test the causal impacts of the tracking and feedback feature on the behavior of the study’s participants. The first, regression discontinuity (RD), leverages the high frequency and longitudinal nature of the outcome observations, coupled with the precisely observed start-date of the interventions. Effectively, the strategy tests for a sudden shift in outcomes when the tracking and feedback features become active.

Equation 1 describes the RD model, where ${y}_{it}$ is the outcome for individual *i* in period *t* and $\text{f}\left({t}_{i}\right)$ is a function that relates period to outcomes. ${T}_{it}$ is a treatment indicator that is equal to one only in periods after the tracking feature has been launched on a participant’s device and zero elsewhere.. ${\varepsilon }_{it}$ is the error term. The interaction between ${T}_{it}$ and $\text{f}\left({t}_{i}\right)$ allows the trends in outcomes to vary before and after the intervention starts.

1 $${y}_{it}=\text{f}\left({t}_{i}\right)+\beta {T}_{it}+\delta {[T}_{it}*\text{f}\left({t}_{i}\right)]+{\varepsilon }_{it}$$

where:

${t}_{i}$=days since the tracking feature launched

$${T}_{it}=\left\{\begin{array}{cc}1& {t}_{i}>0\\ 0& {t}_{i}\le 0\end{array}\right.$$

We first estimated the impacts of the features using a Probit model that includes all the data within four weeks of the treatment (i.e., a total of eight weeks) and a first order relationship between the running variable and the outcome ($\text{f}\left({t}_{i}\right)=\alpha {t}_{i}$). To test the estimates for sensitivity to functional form of the running variable or bandwidth (i.e., the number of days before and after treatment included in the analysis), we re-estimated the impacts using only the data within two weeks of the treatment and a third order relationship between the running variable and the outcome ($\text{f}\left({t}_{i}\right)={\alpha }_{1}{t}_{i}^{1}$+${\alpha }_{2}{t}_{i}^{2}+{\alpha }_{3}{t}_{i}^{3}$).

A difference-in-difference (DiD) model is the second empirical strategy that was used to test for impacts of the treatments. The DiD model cannot be used for testing the impacts of the child feedback feature because all participants had access to the feedback on their phones at the same time and we cannot observe who or when participants accessed it. As discussed in the Study Design and Implementation section, access to the caregiver feedback required that the caregiver manually updated the application on their phone, which generated variation in access treatment date which the updated application also allowed us to observe. While the variation in timing is not experimentally varied, it reflects an arguably arbitrary confluence of factors including which date a caregiver’s phone had power and connectivity, when the project made data credit transfers, when their CHV communicated with them, and their attentiveness to the updating instructions. We tested for differences between those that had updated their application within the first two weeks of its launch and those that had not in the Supplementary Materials and discuss the results in the Results section. The main benefit of the DiD empirical strategy is that it has more power than the RD model.

Equation 2 describes the DiD model, where ${y}_{it}$ is the outcome for individual *i* in period *t*, ${\mu }_{i}$ is a vector of individual fixed effects, ${\alpha }_{t}$ is a vector of period fixed effects, ${T}_{it}$ is an indicator that individual *i* is treated in period *t*, $\beta$ is the estimate of the impact of the treatment on *y*, and ${\varepsilon }_{it}$ is the error. We estimated Eq. 2 using ordinary least squares.

2 $${y}_{it}={\mu }_{i}+{\alpha }_{t}+\beta {T}_{it}+{\varepsilon }_{it}$$

The standard two-way fixed effects (TWFE) model, which is the workhorse for 2-period DiD scenarios, can produce biased estimates in many period models with staggered treatment timing if the impacts are heterogenous across time or individuals [26–29]. We tested for such heterogeneity in the Supplementary Material and presented the results of the TWFE model in the context of those tests for heterogeneity.

Throughout the study, CHVs collected monthly data from the participation caregivers and children on the same indicators being tracked in the app. We ran the same set of analyses as those performed on the caregivers’ data, using the CHVs’ data. Because the CHVs collected data monthly, these additional analyses were performed using a monthly timestep (rather than daily as with the caregiver-collected data). We anticipated less precise estimates using the CHV’s data because there were fewer observations—there was a maximum of 13 observations of each caregiver and child in the CHVs’ data compared to a maximum of 397 observations in the caregivers’ data.

### Tools, data and code

Paper versions of the survey tools used by the participants (caregivers and CHVs) of this study can be found at this link. Ethical clearance was not obtained for placing the data into the public domain; researchers can request access to a partially anonymized version of the data from the lead author. All statistical analyses were performed using STATA-SE version 18.0 (StataCorp LLC, 4905 Lakeway Drive, College Station, Texas 77845 USA). The code used to perform the data processing and analysis described in this manuscript are available at this link.

## Results

The top section of Table 1 provides the summary statistics of the participants at baseline. All 189 caregivers were female and 53% of the participating children were female. Their average ages were 27.4 years and 21.3 months, respectively. About half of the caregivers had received any formal education and could read, and only 11% had used a smartphone.

**Table 1:** Summary statistics of main outcome variables

	N	Mean	Std. Dev	Min	Max
**Demographics at baseline**					
Caregiver: Age (Years)	189	27.4	7.4	15	60.5
Caregiver: Female	189	100%			
Caregiver: Completed any formal education	189	49%			
Caregiver: Can read	189	48%			
Caregiver: Uses a smartphone	189	11%			
Child: Age (months)	189	21.3	10.7	6	47
Child: Gender is female	189	53%			
**Outcomes**					
Children: Minimum Dietary Diversity	31,353	0.58	0.49	0	1
Caregiver: Animal Source Foods	25,570	0.72	0.45	0	1
Caregiver: Dark Green Leafy Vegetables	25,570	0.54	0.50	0	1
Caregiver: Orange Fleshed Fruits and Vegetables	25,570	0.30	0.46	0	1

The bottom section of Table 1 includes the pooled summary statistics across all submissions for the four outcomes of interest. The statistics show that participating children met the minimum dietary diversity threshold in 58% of observations, the caregivers ate *animal source foods* and *dark green leafy vegetables* in more than half of the observations, and *orange fleshed fruits and vegetables* in about a third of the observations.

While active, the caregivers accessed the feedback features an average of 18 times per month.^4^

### Minimum Dietary Diversity for Children (MDD-C)

**Fig. 3:**
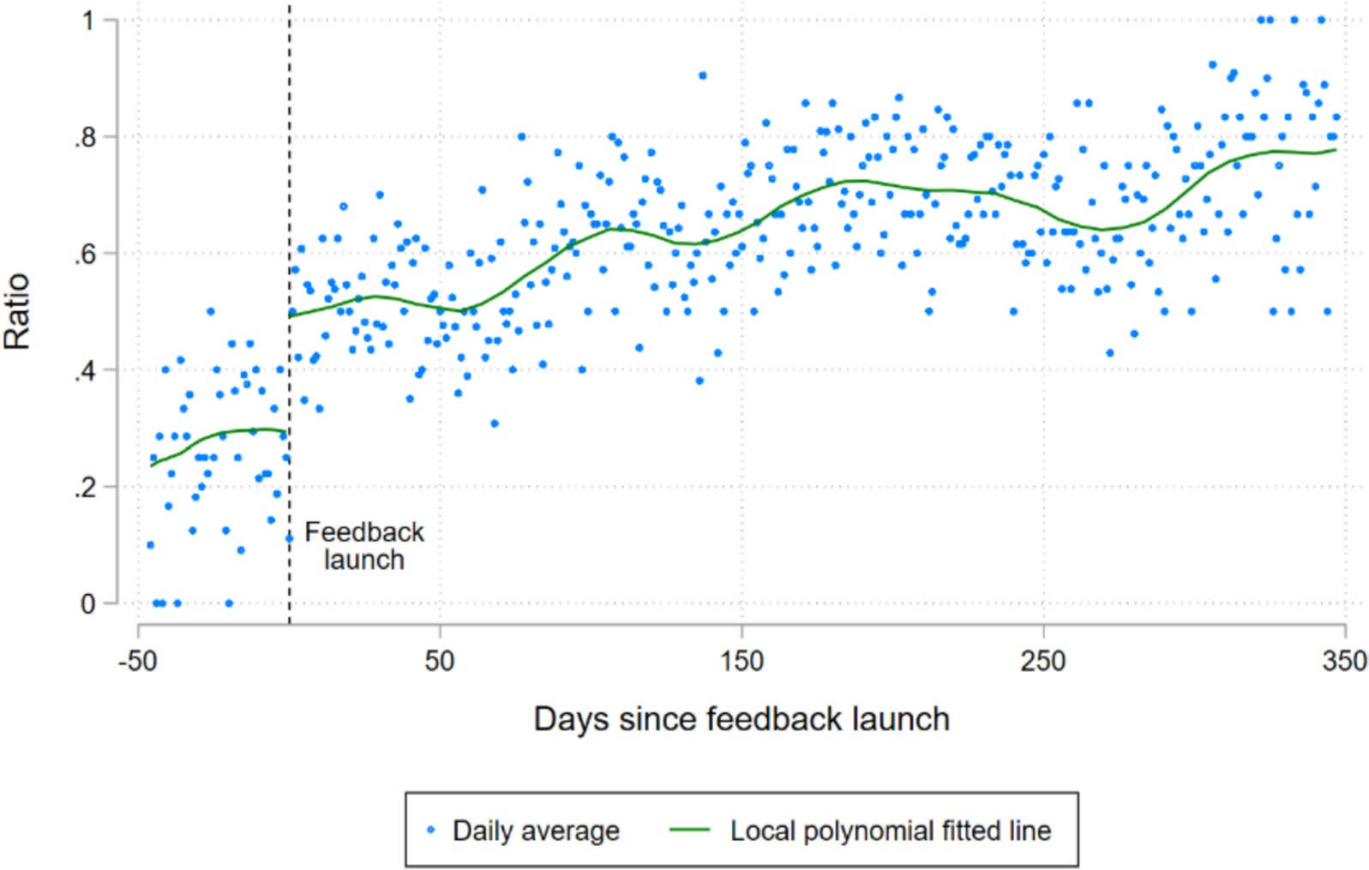
The dots represent the daily ratio of children that met the MDD-C threshold before and after the feedback feature was activated

Figure 3 illustrates the daily ratio of children meeting the MDD-C threshold across time, both before and after the feedback feature was launched.^5^ The figure includes fitted lines from a local polynomial regression, for before and after the launch of the feature. There was an apparent “jump” of about 20 percentage points in the likelihood of a child meeting the MDD-C threshold when the feature launched.

Table 2 includes estimates of the average marginal effects (AME) of the feedback feature on the MDD-C estimated using a Probit model. The models M1 and M2 were estimated using the caregiver collected data. M1 is our primary analysis and includes a linear control for time and observations within 28 days of the treatment. The feature had an average impact of increasing the likelihood of meeting the MDD-C threshold by 23.6% (*p*-value < 0.001). M2 tested if those findings are robust to a more flexible relationship between time and the outcome, and a smaller bandwidth of 14 days. It finds an average estimated effect of 32.5% (*p*-value = 0.055).

**Table 2:** Average marginal impacts of the feedback feature on MDD-C as recorded by caregivers (M1, M2) and by CHVs (M3, M4)

	**Caregiver data**		**CHV data**	
	M1	M2	M3	M4
Impact of feature launch	0.236***	0.325*	0.566***	0.298
	(0.0723)	(0.169)	(0.193)	(0.271)
Observations	971	505	371	215
Polynomial order	1	3	1	1
Bandwidth	28 days	14 days	4 visits^a^	2 visits
N used before launch	303	169	75	75
N used after launch	631	299	296	140

^a^While four post-treatment visits are included in the analysis, there are only two visits available before the treatment. Caregiver-clustered and robust standard errors in parentheses. *** *p* < 0.01, ** *p* < 0.05, * *p* < 0.1

While these findings provide evidence of exactly the type of behavioral changes that the features were developed to generate, there is a possibility that they reflect a change in the caregiver’s recording of consumption that is not actually reflected in the children’s consumption. As a form of verification, we also tested for impacts in the data collected by CHVs from the caregivers on the caregivers’ consumption and that of their child using 24-h recall. Note that the CHVs collected data at monthly intervals, which means that there are only two observations per child before treatment. Therefore, estimating the impacts while simultaneously controlling for higher order polynomial controls was not possible. Models M3 and M4 were estimated using the CHV data, controlling for a linear relationship between time and the outcome. M3 includes the two pre-treatment visits and four post-treatment visits. The estimated AME are 0.566 (*p*-value = 0.003). M4 tested for robustness of this validation exercise by restricting the post-treatment analysis to the two periods after treatment and estimated the AME at 0.298, although the estimates were not statistically significantly different from zero. This loss of statistical significance is not surprising, given the loss of power due to the smaller sample. While this last estimate is not statistically significant, the point estimates across the four models place the impact of the feature as increasing the ratio of children that met the MDD-C threshold at 23%−57%.

### Targeted food group consumption among caregivers

**Fig. 4:**
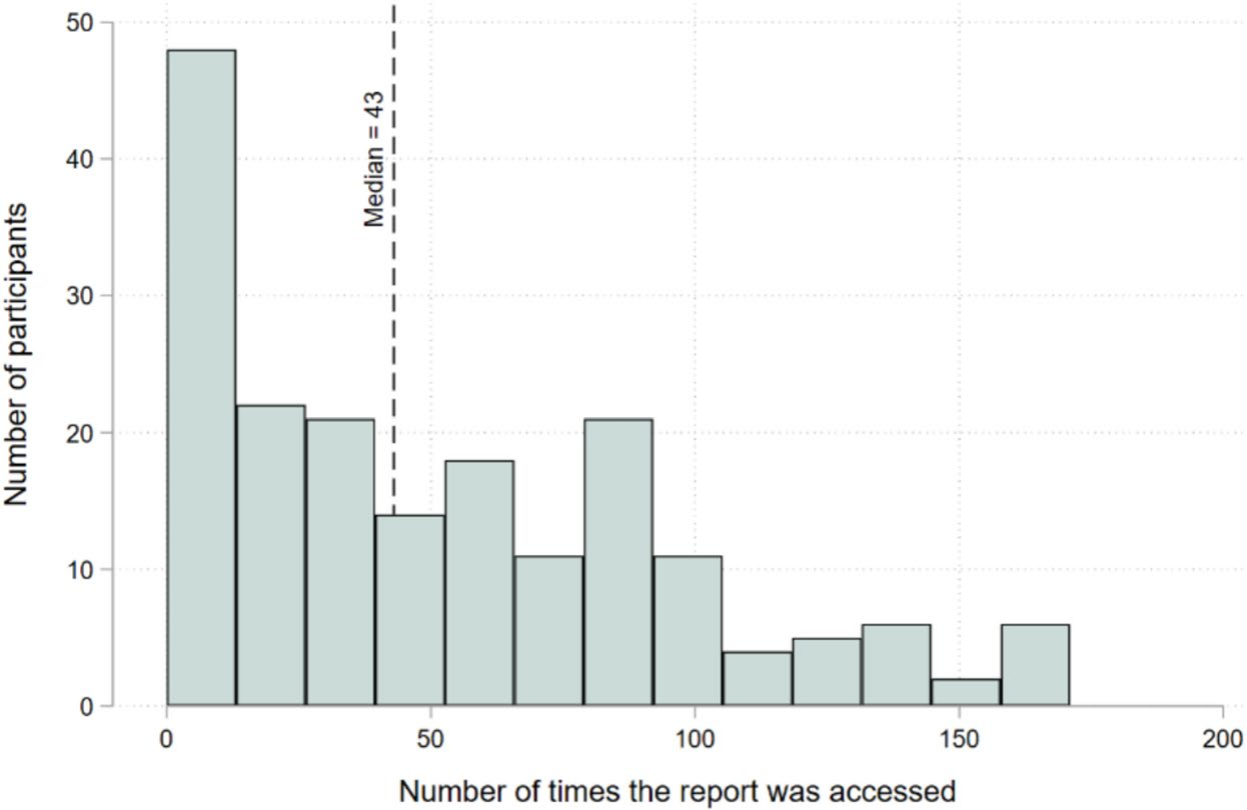
The number of times that each participant accessed a caregiver feedback report during the study period

We now turn to the second feature, which provided feedback to the caregiver on her own consumption. Figure 4 illustrates the number of times each caregiver accessed this second feature over the project period. A total of 24 participants never accessed the tracking feature and the median number of times that the feature was accessed is 43 (Fig. 4).

In the following section, we present the RD impact estimates of this second feature, followed by the DiD impact estimates as robustness checks.

#### Regression discontinuity estimates

**Fig. 5:**
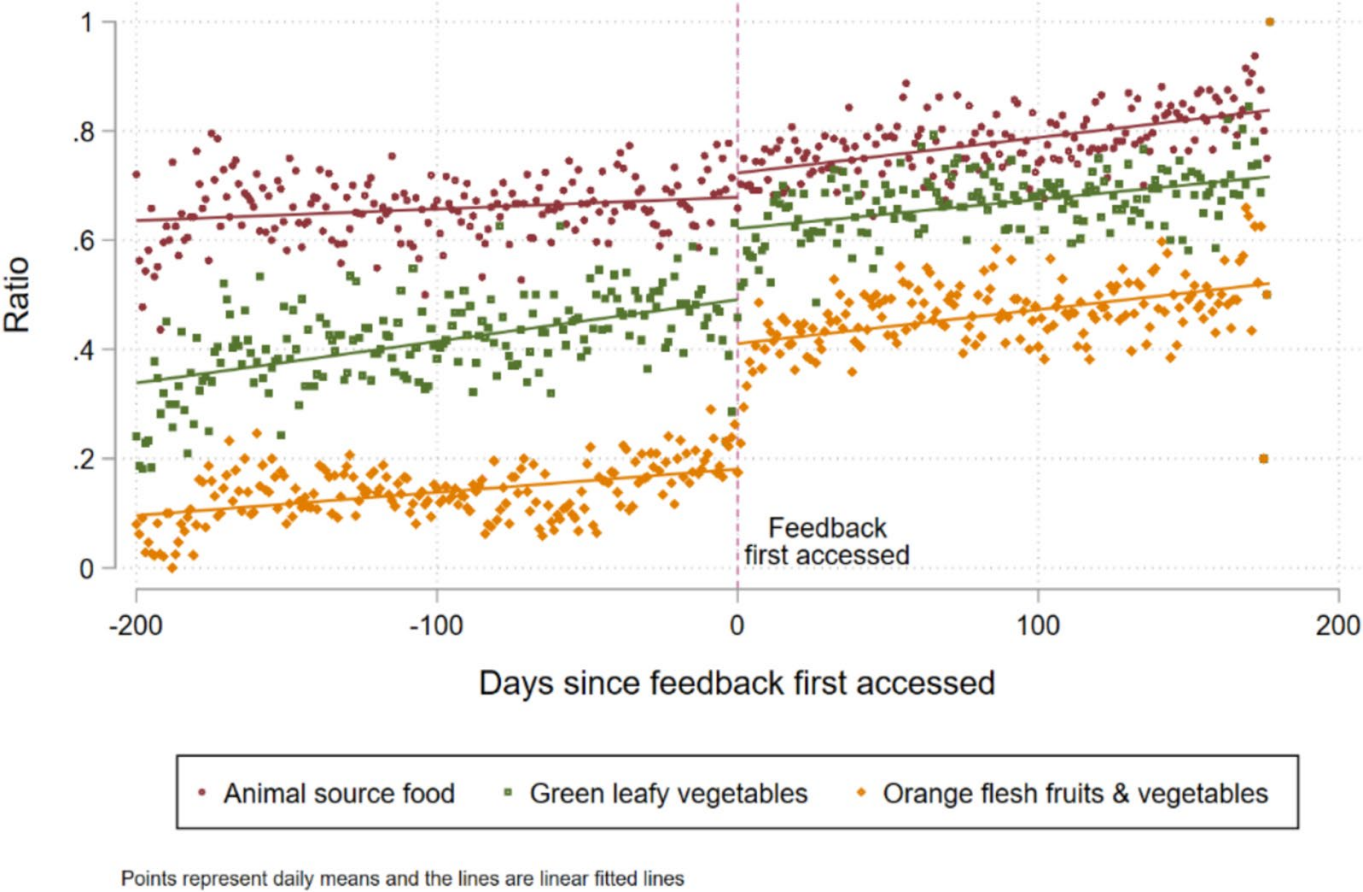
Ratio of caregivers that had consumed foods from each group over the previous 24 h

For this analysis, we developed a caregiver-specific version of the running variable—days since the feature was first accessed by that caregiver—which is equal to zero on the day that the caregiver first accessed the tracking feature. Figure 5 illustrates the three binary outcome variables, each that is equal to one if the caregiver recorded eating a food from each respective food group in the last 24 h and equal to zero if not, along the running variable. We also included linear fitted lines to illustrate the possible shift in consumption as caregivers started accessing the feedback. Note that while there does seem to be a shift, it is smaller than the shift that was apparent in Fig. 3 in MDD-C.

We then ran the same set of analyses as we conducted earlier on MDD-C, this time on each of the three caregiver outcomes. The first model (M1, Table 3), which was our primary test of impacts and used the data recorded by the caregivers, showed positive and statistically significant average treatment effects of 11.5% and 15.0% increases of consuming *dark green leafy vegetables* and *orange fleshed fruits & vegetables*, respectively, but no impacts on consumption of *animal source food*. In the robustness check in which the bandwidth was reduced and higher order polynomials of time were included, the estimates became much smaller and lost statistical significance (M2, Table 3). It may be that estimating a third order polynomial with 14 days of data is a weak empirical strategy.

**Table 3:** The estimated average marginal effects of the caregiver feedback on the likelihood of consuming food groups in the caregiver data

	**Caregiver data**		**CHV data**	
Impact on Consumption of:	M1	M2	M3	M4
Animal Source Foods	0.013	0.049	0.0447	0.151**
	(0.0360)	(0.0629)	(0.0314)	(0.0636)
Dark Green Leafy Vegetables	0.115***	0.015	0.0870**	0.0943
	(0.0385)	(0.0764)	(0.0443)	(0.0788)
Orange Fleshed Fruits & Vegetables	0.150***	−0.001	0.0494	0.0131
	(0.0333)	(0.0704)	(0.0356)	(0.0781)
Model statistics				
Observations	23,819	23,819	1876	718
Polynomial order	1	3	1	1
Bandwidth	28 days	14 days	4 visits	2 visits
N before launch	14,199	465	858	517
N after launch	10,889	1,069	1018	689

Each row and column represent the coefficient estimates from a separate analysis. All the analysis in a column use the same model and therefore have the same model statistics, which are presented once at the bottom of the table. Caregiver-clustered and robust standard errors in parentheses. *** *p* < 0.01, ** *p* < 0.05, * *p* < 0.1

Models 3 and 4 (M3 and M4 in Table 3) provide the estimates from an analysis that used the data collected by CHV to test if similar impacts were evident there. In common with the results that used the caregiver collected data, these analyses provide evidence of positive impacts but the statistical significance was sensitive to the specification strategy.^6^

#### Difference in difference estimates

**Fig. 6:**
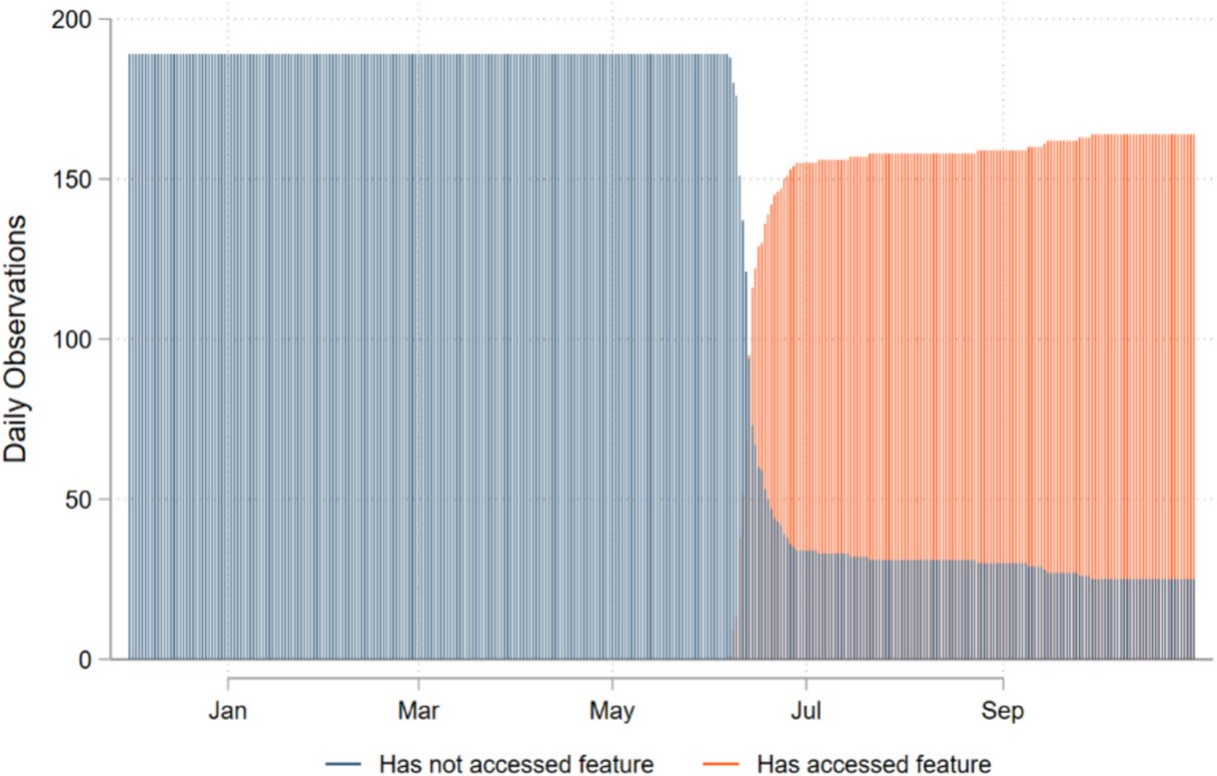
The number of caregivers out of 189 that had not ever accessed or that had accessed the caregiver feedback feature, calculated each day over the project period

The impacts of accessing the caregiver feedback were tested using the DiD model. Figure 6 illustrates the sample size across time between the two groups. Importantly for the DiD empirical strategy, there continued to be non-adopters after the feature was launched.

As stated earlier, the DiD model should have more power than the RD model, but there are several concerns related to parallel pre-treatment trends and heterogenous impacts that need to be considered. We examined those concerns in the Supplementary Material. Analysis for differences between those that accessed the caregiver feedback feature within the first two weeks (78% of caregivers) after the update was launched and those that did not (22% of caregivers), are provided in the Supplementary Materials. The analysis finds that at baseline, those that updated in the first two weeks were about 2 years younger on average (*P*-value = 0.07) than the other group but that there were no differences in education, literacy or access to smartphones (Supplementary Table 1). An analysis of caregivers’ consumption during the first 180 days of the project, before the caregiver’s feedback feature was launched, found no difference in the consumption trends for any of the three consumption outcomes (Supplementary Table 2). The analysis for heterogenous impacts are discussed after the main regression results.

The DiD estimates using the caregiver-measured and the CHV-measured data are provided in Table 4. Similar to the RD results, the coefficient estimates generated from the caregiver measured data indicated a positive change in consumption behavior across all three food groups, but in this case, all estimates were highly statistically significant. Those positive changes are also reflected in the point estimates made from the CHV-measurements, although there was a loss of statistical significance for *dark green leafy vegetables*.

**Table 4:** Difference in difference estimates of the impact of the feedback feature on caregiver consumption

	Animal Source Foods	Dark Green Leafy Vegetables	Orange Flesh Fruits & Vegetables
**Caregiver measured**			
Impact of feature	0.0867***	0.159***	0.233***
	(0.0211)	(0.0285)	(0.0346)
Observations	21,995	21,995	21,995
R-squared	0.353	0.395	0.439
Individual Fixed effects	Yes	Yes	Yes
Daily Fixed effects	Yes	Yes	Yes
**CHV measured**			
Impact of feature	0.117***	0.0549	0.106**
	(0.0364)	(0.0510)	(0.0409)
Observations	1,638	1,638	1,638
R-squared	0.468	0.445	0.329
Individual Fixed effects	Yes	Yes	Yes
Monthly Fixed effects	Yes	Yes	Yes

Standard errors are clustered at the individual level and reported in parentheses under their respective coefficient estimate. *** *p* < 0.01, ** *p* < 0.05, * *p* < 0.1

Heterogeneity in impacts can cause bias in the TWFE estimates. We tested for heterogenous impacts in the caregiver data across the duration of treatment and individual, using the processes suggested by Jakiela [29]. That analysis, which is provided in the Supplementary Material, found that the RD estimates were unstable if analysis was restricted to a short period before and after the treatments launch, but stabilized once about 50 pre- and post-treatment days were included. Importantly, there is no evidence that heterogenous impacts biased the estimates presented in Table 4; indeed, the estimates were extremely stable across treatment period and treatment individual.

**Fig. 7:**
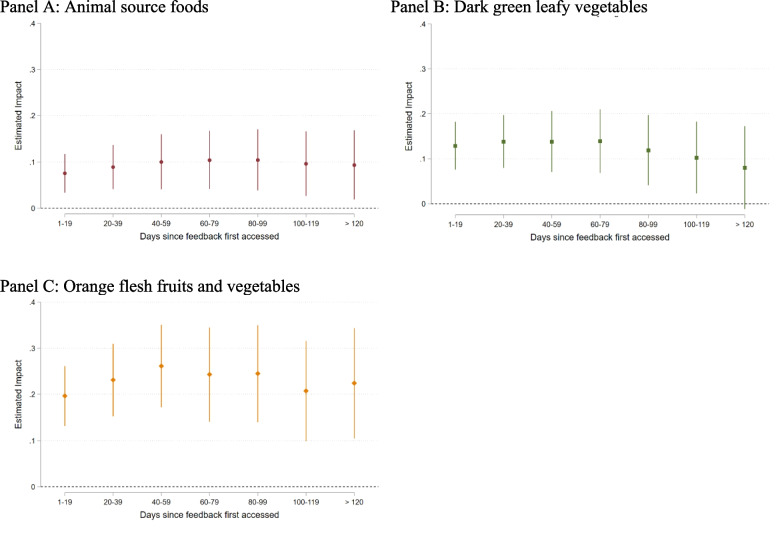
Estimated persistence of the impact of the feedback feature on caregiver consumption of animal source foods (Panel **A**), dark green leafy vegetables (Panel **B**), and orange flesh fruits and vegetables (Panel **C**)

An additional advantage of the DiD empirical approach is that we can test for the persistence of impacts over time. To do this, we change the treatment status variable to be a series of binned treatment statuses based on the duration of treatment and then re-estimate the DID model. There were insufficient observations in the CHV data for this analysis. The results using the caregiver data are presented in Fig. 7. They show that the impacts of the treatment persisted throughout the study. Indeed, there was very little indication that the impacts were declining, even 120 days after the caregivers first started accessing the feedback on their consumption, although there was a small loss in precision and a very gradual decreasing trend in the impacts on consumption of dark green leafy vegetables (Fig. 7, Panel B).

## Discussion

This study combined nutrition education with nutrition tracking and provided participants with tailored feedback on their consumption. We found strong evidence that the consumption of the children participating in the project changed in response to the tailored advice; in both the caregiver’s self-recorded measurements and those submitted by CHVs, the children were at least 23 percentage points more likely to meet the minimum dietary diversity threshold. We also found strong evidence in both the measurements made by caregivers and by CHVs that the caregivers changed their own consumption patterns in response to the tailored advice on their own consumption patterns and that those changes persisted for the remaining months of the project.

The main limitation of our study is that we did not collect information on intermediary outcomes that would have allowed us to unpack the mechanism by which the tracking and feedback feature impacted behavior. Those impacts could have been caused by improved awareness of the recommendations (education), by improving the participants’ own tracking of their behavior (reduced burden of tracking), or by proving tailored and actionable feedback. A second limitation is that we did not have stronger validation data. Caregivers may have adjusted their reporting in the application and to the CHVs in response to the feedback features without adjusting consumption behavior. It would have strengthened our findings if we had collected measurements based on third party (e.g. enumerator-collected) observations as well.

## Conclusion

Our results indicate that there is considerable promise for improving consumption by providing tailored feedback through the approaches tested by this research, especially among the millions of people living in remote locations with little access to health education and services. While ours is not the first study to show that tailored feedback on consumption can change consumption behavior, we are the first to combine self-recording and tailored feedback in a low-income and remote setting with high rates of malnutrition. That remoteness, poor access to services, and malnutrition often coincide is not coincidence; it is much more difficult and expensive to provide such services to remote locations. Those relationships also imply that the low-cost and light-touch approaches tested here are likely to hold the greatest value among those living in remote areas and/or that are often left out of existing health education and monitoring services for other reasons.

## Supplementary Information

Supplementary Material 1.

Supplementary Material 2.

## Data Availability

The datasets used during the current study are available from the corresponding author Nathan Jensen at njensen@ed.ac.uk on reasonable request.

## Abbreviations

CHV: Community Health Volunteer
DiD: Difference in Difference
ILRI: International Livestock Research Institute
MDD-C: Minimum Dietary Diversity Score for Children
MUAC: Mid-upper arm circumference
RD: Regression discontinuity
TWFE: Two-way fixed effects

## Footnotes

1 Previous research showed that the accuracy of the data recorded by caregivers through this application was similar to that of data collected by enumerators in similar settings, and that it offered a lower-cost approach to collecting high-frequency data than other feasible options [22].

2 All submissions were completely voluntary and participants could submit reports as much (up to the max) or as little as they liked. The incentive was provided on a per report basis. Each 24-h period, caregivers could submit up to one report on their own consumption and one report on their child’s. Every 6 days, they could submit a report on their child’s mid-upper arm circumference (MUAC). They received about USD0.3 per submission. USD5 of airtime and data was remotely loaded onto their phones each month to ensure that the data was synched without challenges.

3 As an example, the audio message for the middle panel states in the Samburu language “Hello Mama, your child is recommended to consume at least 4 food groups in a day. Below you will see an image representing consumption, on the side you will either see green tick or red x, green indicates that your child is consuming foods as recommended. Red x indicates that your child is not consuming foods as recommended, if you see red kindly talk to the CHV.”.

4 The application was updated on February 26 of 2020, about 4 months after the project started, to track when caregiver accessed to the feedback processes. Between February 26 and December 31, 2020, the caregivers accessed the feedback processes a total of 34,057 times.

5 There is a period between the feature becoming active in December 15, and a set of trainings that took place in December 19, 20, 21, and 23, during which the feature was active but the caregivers had not been trained nor had their attention drawn to the new feature. Because we cannot observe if the caregivers were aware or used the feature during this period, the “between” days have been dropped completely from the dataset. The result is that December 14 th represents the day before activation (t = -1), the period between activation and training is condensed into a single day (t = 0) and the day after training is t = 1.

6 Here we note that Table 3 presents twelve analyses without adjusting for multiple hypothesis testing. We perceive M1 to be the primary test for impacts, M2 to be a robustness check, and M3 and M4 represent an effort to validate the main findings using alternative, potentially more trustworthy data.
