# Supplementary material for: Impacts of consumption tracking and tailored feedback on meeting nutritional recommendations: a longitudinal regression discontinuity study
Source: Nutr J. 2025 May 23;24:85. doi: 10.1186/s12937-025-01149-x (PMC12100978; doi:10.1186/s12937-025-01149-x)

Supplementary Material

Impacts of consumption tracking and tailored feedback on meeting nutritional recommendations: A longitudinal regression discontinuity study

Tests for differences between standard adopters and late/non-adopters

Unlike the launch of the child feedback feature, the caregivers had to update their application to obtain access to the caregiver's feedback feature. Once available on 1 June 2020, this updating process was optional and could be done any time the caregiver had connectivity. While most participants updated their application within the first two weeks, some took much longer and quite a few never updated at all. This user-determined timing creates a risk that caregivers' attributes that effected the timing of their application updating also effected their consumption, which would violate the assumptions of causal identification using the DiD approach. We test for this in two ways. In both analyses, we define standard adopters as those that updated their application within the first two weeks of its launch. The remaining, which we call late/non-adopters here, are composed of 17 participants (9% of our sample) that updated after those two weeks and 25 participants (13% of our sample) that never updated their applications.

First, we test for differences between the two groups—standard adopters and late/non-adopters—in demographic information collected at baseline (Table 1). Here we find that the late/non-adopters are older by an average of 2.2 years (p-value=0.07), but no differences in any of the other variables measured. This finding is slightly concerning but the results on reading, education and smart-phone access do not indicate socio-economic, education or access differences, which would be much more concerning.

Supplementary Table 1: Difference in baseline characteristics between standard and late/non-adopters

	**Standard adopters**	**Late/non-adopters**	**Pairwise t-test**
	Mean (SE)	Mean (SE)	Mean difference
Age of participant (years)	26.854	29.036	-2.182*
	(0.575)	(1.385)	
Participant can read	0.483	0.476	0.007
	(0.041)	(0.078)	
Participant has had any formal education	0.476	0.500	-0.024
	(0.041)	(0.078)	
Participant has access to a smartphone	0.102	0.143	-0.041
	(0.025)	(0.055)	
Number of participants	147	42	

Standard errors in parenthesis. *** p<0.01, ** p<0.05, * p<0.1

Our second test is to use data from the first half of the study to test if the two groups have similar consumption treads before the feature was launched. Unbiased DiD estimates of causal impacts require that the pre-treatment trends be parallel or any difference needs to by convincingly controlled for. To test for differences in pre-treatment trends, we individually regress our three outcomes onto a time variable using a probit model, allowing for variation in slope and intercept between the two groups. Supplementary Table 2 provides these estimates, where our main variable of interest is the estimates on the differences in slopes, which is labeled *Difference in trends between standard and late/non-adopters*. In this case, there is no statistical difference in the trends before the feature was launched, which supports the validity of the DiD model for estimating causal effects.

Supplementary Table 2: Estimates from a Probit regression testing for differences in consumption trends between standard and late/non-adopters before the caregiver feature launched.

	Animal Source Foods	Green Leafy Vegetables	Orange Fleshed Fruits & Vegetables
Trend among standard adopters	0.000141	0.00149***	0.00142**
	(0.000562)	(0.000526)	(0.000685)
Difference in trends between standard and late/non-adopters	-0.000929	0.00208	-0.000952
	(0.00130)	(0.00151)	(0.00159)
Intercept among standard adopters	0.392***	-0.331***	-1.221***
	(0.0794)	(0.0801)	(0.0969)
Difference in intercept between standard and late/non-adopters	-0.000929	0.00208	-0.000952
	(0.00130)	(0.00151)	(0.00159)
Observations	11,949	11,949	11,949

Standard errors, which are clustered at caregiver level, in parentheses. *** p<0.01, ** p<0.05, * p<0.1

Tests for heterogeneity in Differences in Differences (DiD) estimates

To test for heterogeneity in impacts across treatment periods, we run the analysis using the caregiver collected data for each of the three food groups, restricting the number of days after the treatment started from 10 to 150—the maximum number of post-treatment days in the database is 147—moving in units of 20 days. The results are presented in Supplementary Figure 1. In all cases, the estimates are generated using ordinary least squares (OLS) of the model described in Equation 1, and the standard errors are clustered at the individual-level. For all three food groups, the estimated impacts are largest and least precisely estimated immediately after the treatment, but then stabilize in magnitude and improve in precision when at least 50 post-treatment days are included in the analysis. Our estimates in the main text of the manuscript use the full set of observed post-treatment days (i.e., the estimates found on the right side of each panel in Supplementary Figure 1) and this analysis reveals none of the heterogeneity across treatment periods that would suggest bias in our estimates.

Supplementary Figure 1. Difference in differences estimates of the impact of feedback on consumption of *animal source foods* (Panel A), *dark green leafy vegetables* (Panel B), and *orange fleshed fruits and vegetables* (Panel C). The figures illustrate the estimates associated with varying the number of post-treatment days from 10 to 150 days.

Panel A: Animal source foods

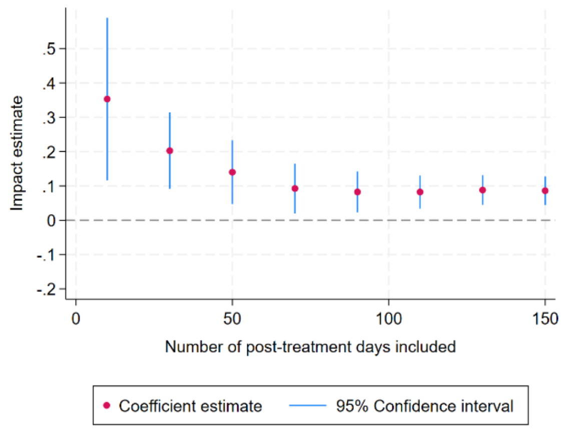

Panel B: Dark green leafy vegetables

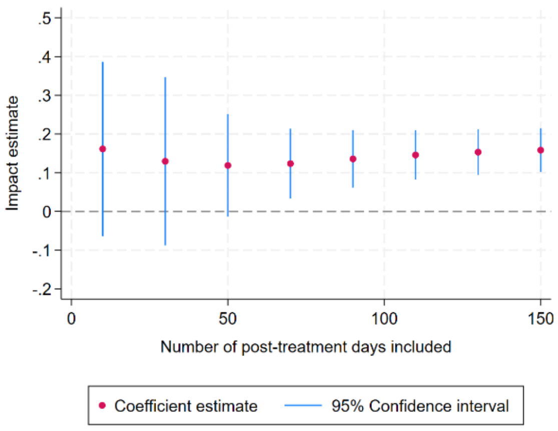

Panel C: Orange flesh fruits and vegetables

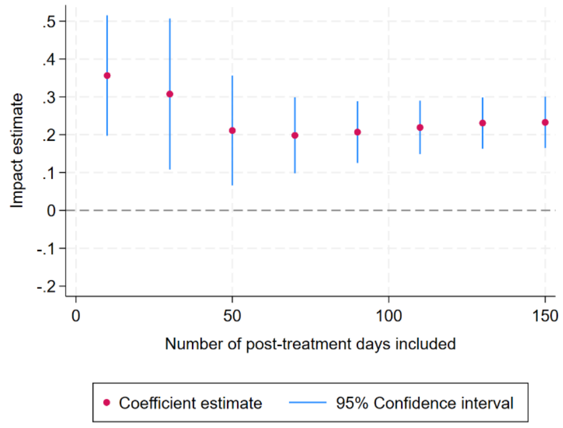

We then test for variation in the estimates across individuals. To do so, we re-estimate the DiD model on each of the three food groups using only a random draw of 60% of the individuals. In this analysis, all post-treatment days are included. We repeat this process 200 times, each time re-drawing the subsample. Note that the estimates generated from this sub-sampling approach are likely to be less precise than those estimated with the full sample because the sample size is only 60% of the total sample size. The estimates are illustrated by the three panels in Supplementary Figure 2, where the estimates have been ordered by magnitude for easier interpretation.

The estimates across the 200 subsamples are quite similar across the distribution of estimates all three food groups; of the 200 estimates for each food group, less than 10% of estimates are statistically distinguishable from each other at the 10% level (i.e., largest 5% of the estimates are statistically distinguishable from the smallest 5%). Less than 4% of the resampled estimates from each group are statistically significantly different from the estimates provided in Table 6 from the full sample of participants.

Supplementary Figure 2. Distribution of difference in differences estimates of the impact of feedback on consumption of *animal source foods* (Panel A), *dark green leafy vegetables* (Panel B), and *orange fleshed fruits and vegetables* (Panel C). Each estimate is generated by performing the TWFE process on a randomly drawn subsample of 60% of the individuals included in the original sample.

Panel A: Animal source foods

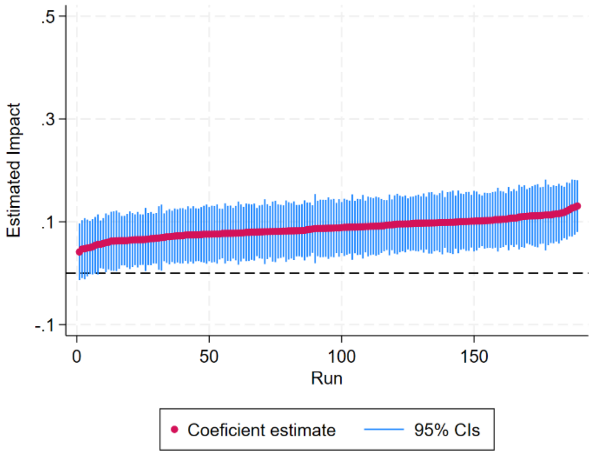

Panel B: Dark green leafy vegetables

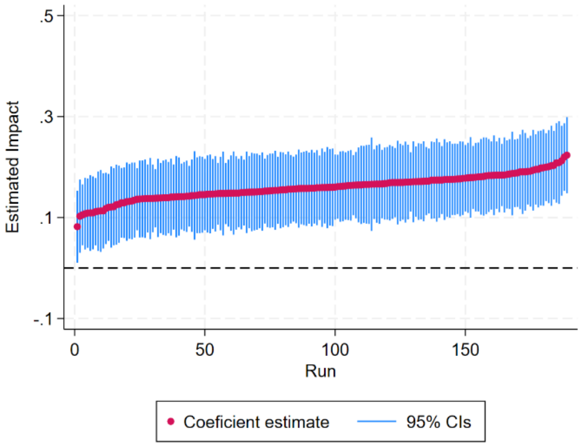

Panel C: Orange flesh fruits and vegetables

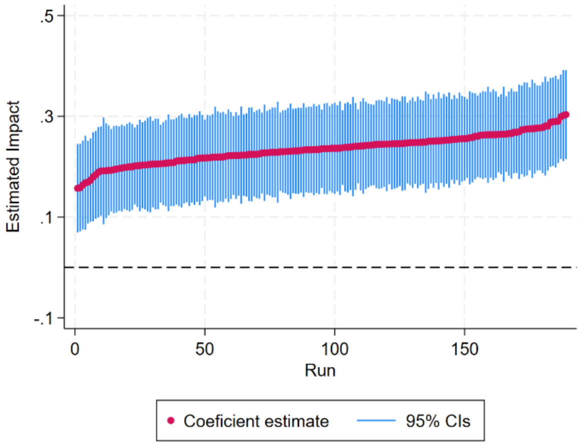

Supplement: Supplementary file 1 — Supplementary Material 1. [file 12937_2025_1149_MOESM1_ESM.pdf]
