# Supplementary material for: Impacts of consumption tracking and tailored feedback on meeting nutritional recommendations: a longitudinal regression discontinuity study
Source: Nutr J. 2025 May 23;24:85. doi: 10.1186/s12937-025-01149-x (PMC12100978; doi:10.1186/s12937-025-01149-x)
Supplement: Supplementary file 2 — Supplementary Material 2. [file 12937_2025_1149_MOESM2_ESM.pdf]

Consent

Enumerator: please read the following consent script, obtain the consent and signature

I am here on behalf of the International Livestock Research Institute (ILRI), an institute doing agricultural and livestock research in Africa and around the world. We are conducting a study to develop a tool by which caregivers can collect, submit, and track nutrition and health information of themselves and the children that they care for. To develop this tool, we will be working with caregivers to develop and improve the tool. In short, we will be asking caregivers to record the types of food that they and one of their children has eaten in the last 24 hours, any clinical symptoms experienced by the caregiver or the child in the last 24 hours, a few questions on food security, and for you to measure and record your child's mid-upper arm circumference. We will also ask you to take a photo of your child's face and upper arm.

This information will be recorded first using a paper survey, then later as a smartphone application. The survey is completed by ticking small images and does not require literacy. We will be providing participating caregivers with training, use of a smart phone, and a solar charge means. The smart phone application will also provide you with information benchmarking past and current nutritional status.

In total, we expect that the project will run for about 12 months. We are hoping that caregivers will complete the survey about every week during that period. Caregivers will be rewarded 50KSH for each submission and an will be reimbursed for data bundles should they be required.

If you agree to participate, the CHV will register you and one of the children that you take care of as participants. The child should be the youngest child that is over 6 months old. The CHV will also visit your house periodically to provide you with help should you have problems with the survey application and to also collect similar information from you.

You may ask questions now or anytime during the project. All the information you give will be strictly anonymous, which means your name will not be associated with any of your responses or given to anyone outside our project. If you would rather not participate in the project, you may opt-out of this project now or at any time that you wish. You may also agree to be registered today, but opt out of the project at any time.

Do you have any questions?

Do you agree to participate in this study? ☐ YES ☐ NO

Do you agree that a child for whom you are the guardian and main caregiver and that is between 6 and 52 months old will participate in this study? ☐ YES ☐ NO

There are some additional activities we'd like to see if you'd be interested to participate in. Feel free to agree or disagree, as this would not compromise your participation in the study.

We may like to return in after the project to re-interview the household. Do you agree to be contacted again to invite you to participate in future interviews? ☐ YES ☐ NO

We would also like to invite you to participate in other research activities, including shorter surveys and games, over the next several years. Do you agree to be contacted in the future to invite you to participate in such additional activities? ☐ YES ☐ NO

Your cooperation is greatly appreciated, as it will help us to understand health and nutrition among pastoral households.

May we proceed with the interview?

Collect signature

If you have any questions throughout the study, you can contact Dr. Nathan Jensen at 071-267-9990, the leader of this project and a scientist at ILRI.

If you have any complaints or questions about the interview, please call ILRI's research compliance office at 020-422-3375.
